# EHMTI-0140. The potential role of levetiracetam in migraine treatment: an animal study

**DOI:** 10.1186/1129-2377-15-S1-F30

**Published:** 2014-09-18

**Authors:** YF Wang, JC Yen, LS Kao, JL Fuh, SJ Wang

**Affiliations:** 1 Department of Neurology, Taipei Veterans General Hospital, Taipei, Taiwan; 2 School of Medicine, National Yang-Ming University, Taipei, Taiwan

## Introduction

Cortical spreading depression (CSD) is one of the most widely used animal models of migraine. Whether levetiracetam (LEV), like other antiepileptic drugs, has a role in the treatment of migraine remains uncertain.

## Aim

To investigate the potential of LEV in the treatment of migraine using a rat model of CSD.

## Method

Male Sprague-Dawley rats were used. The effects of acute (3 day) and chronic (28 days) treatment with vehicle, LEV 200mg/kg/d, and LEV 400mg/kg/d on CSD susceptibility were examined. Drugs were given as daily intraperitoneal injections. After completion of drug treatment, CSD was elicited by placing a cotton ball soaked with 1M KCl onto the occipital cortex, and was recorded for 2 hours by placing a glass microelectrode into the frontal cortex.

## Results

In the acute treatment experiment, rats receiving LEV 400mg/kg/d (8.4±1.0) had fewer CSDs per hour than those receiving vehicle (12.9±1.7, p<0.001) and LEV 200mg/kg/d (12.5±1.2, p<0.001). In the chronic treatment experiment, rats receiving LEV 400mg/kg/d (11.4±0.6) had fewer hourly CSD events than those receiving vehicle (14.3±0.3, P<0.001) and LEV 200mg/kg/d (13.6±0.4, p<0.001), and rats treated LEV 200mg/kg/d had less CSDs than those in the vehicle group (p=0.049).

## Conclusion

LEV had a modest effect on reducing CSD susceptibility at a dose of 400mg/kg/d, and the effects on CSD susceptibility were comparable when administered acutely or chronically.

Conflict of interest.

